# Structural differentiation and collaborative optimization of the spatial network of pension care service in megacity behemoths: a case study of Guangzhou, China

**DOI:** 10.3389/fpubh.2026.1960926

**Published:** 2026-10-19

**Authors:** Lianhua Liu, Liuzhou Lan, Yinglin Li, Ziling Wang

**Affiliations:** 1 Guangzhou Huashang College, Guangzhou, China; 2 Guangzhou International Economics College, Guangzhou, China

**Keywords:** collaborative optimization, megacity, pension care services, spatial network, structural differentiation

## Abstract

**Introduction:**

Against the backdrop of population aging, cross-district coordination and equitable allocation of pension care resources have become increasingly important for improving service accessibility and urban governance in megacities. This study examines spatial disparities and interdistrict connections in pension care services across Guangzhou’s 11 districts, focusing on resource distribution, transportation accessibility, and network structure.

**Methods:**

A multidimensional evaluation framework encompassing institutional care capacity, home- and community-based care capacity, and medical support capacity for pension care was developed. The entropy weighting method was employed to evaluate the comprehensive development level of pension care services in each district. An interdistrict transport impedance index was constructed based on public transportation travel times, and a modified gravity model was applied to estimate the strength of interdistrict connections in pension care services. Social network analysis was subsequently conducted to examine network density, centrality, and core–periphery structures.

**Results:**

The findings reveal substantial spatial disparities and functional differentiation in pension care services across Guangzhou. Transportation accessibility generally decreases from central to peripheral districts. The interdistrict service network exhibits a centralized and hierarchical structure, with Baiyun and Tianhe occupying prominent positions in network connectivity. Liwan, Tianhe, and Baiyun constitute the network’s core area, whereas peripheral districts demonstrate relatively weak interdistrict connections and insufficient coordination.

**Discussion:**

The findings underscore the need to strengthen cross-district coordination and address spatial inequalities in pension care services. Policy priorities include enhancing resource integration and service coordination among core districts, developing secondary pension care service centers, and improving mechanisms for resource sharing, service referral, and digital collaboration in peripheral districts. These measures could contribute to a more balanced, multi-tiered, and interconnected urban pension care service network.

## 1 Introduction

Against the backdrop of an increasingly aging population, whether pension care resources can be rationally distributed and coordinated across regions has become a critical issue for improving the accessibility and equity of urban pension care services. In megacities, urban districts differ significantly in population structure, facility supply, medical resources, and transportation conditions. Pension care services are not confined to a single district but form cross-district connections through institutional admission, medical referrals, community services, and resource sharing. Simply examining the number of pension care facilities or the overall level of service provision within each district can identify spatial differences in resource stocks. Still, it cannot fully explain how the functional influence of these resources may extend across administrative boundaries. In megacities, the spatial effectiveness of pension care resources depends not only on where resources are located, but also on whether districts are connected through transportation networks, service referrals, institutional collaboration, and resource sharing. Districts therefore function not only as containers of pension care resources but also as relational nodes embedded in a broader urban service system. From this perspective, static comparisons of district-level resource endowments need to be complemented by an analysis of potential interdistrict linkages and network positions.

Guangzhou was selected not merely because of its megacity scale, but because it provides an analytically informative setting for examining interdistrict differentiation and relational structures in pension care services. Its 11 administrative districts show marked heterogeneity in population aging, pension care resource endowments, medical service capacity, and public transportation accessibility. At the same time, the city includes traditional central districts, expanding urban areas, and peripheral districts with differentiated functional roles. This coexistence of resource disparities, transport frictions, and spatial-functional differentiation provides a suitable context for examining how differences in district-level service capacity relate to potential interdistrict linkages and network positions. As of 2024, Guangzhou had 2,129,210 registered residents aged 60 and older, accounting for 19.81% of the registered population. The distribution of the older residents across the 11 districts is uneven, with Yuexiu District having the highest number at 351,130 and Nansha District the lowest at 94,582, a range of 256,548. The proportion of older adults differs significantly across districts. Liwan District has a proportion of 31.61% for those aged 60 and over, which is 18.48 percentage points higher than Huangpu District, which has the lowest proportion at 13.13%. The districts exhibit functional differentiation in institutional care capacity, community-based care capacity, and medical support capacity for pension care, resulting in significant spatial imbalance in the overall development of pension care services. For example, the total number of nursing home beds is 57,452, with Baiyun District accounting for 23.13% and Conghua District for only 1.35%. The uneven distribution of pension care resources provides a practical foundation for cross-district resource complementarity and service collaboration. At the same time, the relatively high travel time costs in outlying urban areas may constrain the reach of pension care resources, referrals for professional services, and family visits, resulting in distinct patterns of spatial connectivity in pension care services.

Based on these considerations, this study uses the 11 administrative districts of Guangzhou as units of analysis. We construct an evaluation system across three dimensions—institutional care capacity, home-based and community-based care capacity, and medical support capacity for pension care—to assess the overall development level of pension care services in each district. Public transportation travel time is then introduced to characterize interdistrict spatial resistance, and a modified gravity model is used to estimate potential spatial linkages in pension care services. Social network analysis is further employed to identify the overall network structure and differences in node positions. Accordingly, this study addresses three interrelated research questions: RQ1: What spatial differences exist in the development level of pension care services and transportation accessibility across Guangzhou’s 11 districts? RQ2: What patterns of potential interdistrict pension care service linkages emerge under the joint influence of district-level service capacity and transportation resistance? RQ3: How are these potential interdistrict linkages organized into an overall network, and how do districts differ in terms of centrality and core-periphery position? By linking node capacity, interdistrict potential linkages, and overall network structure within a unified analytical framework, this study extends district-level pension care research from static comparisons of resource endowments toward a relational analysis of potential cross-district connections and structural positions.

## 2 Literature review

### 2.1 Theoretical foundations and integrated analytical framework

The spatial allocation of pension care services involves questions of resource equity, supply–demand fit, and interregional coordination. Spatial justice, supply–demand matching, and network governance therefore offer three complementary perspectives on the spatial structure of pension care services in megacities. Spatial justice concerns whether older adults in different areas have equitable opportunities to access services. Previous studies incorporated multilevel medical facilities and community-based care facilities into an assessment of healthcare accessibility for older adults. They showed that similar facility numbers do not necessarily imply equal access to services (1). Recent studies have incorporated the joint importance of local proximity and socio-spatial equity through the concept of “super-accessibility” (2). Marston et al. extended the age-friendly city framework to include smart homes, digital platforms, and e-health services (3), suggesting that the spatial organization of pension care is shaped by the interaction of physical space, social relations, and digital connectivity. The supply–demand matching perspective emphasizes that pension care resource allocation should respond to differences in population needs and local service capacity. Wang and Zhou (4) argued that pension care planning should take account of health status, changes in service demand, and the location of urban service centers rather than rely solely on the size of the older population. Regional differences in pension care allocation from the perspective of supply–demand coordination (5). Subsequent studies further showed that resource input, demographic equity, geographical equity, and allocation efficiency do not follow a simple linear relationship (6–8). Increasing the number of facilities alone, therefore, does not necessarily improve spatial equity in pension care services. Network governance shifts the focus from how many resources a district possesses to how districts are connected. Existing research suggests that older adults’ service needs are embedded in family, community, and broader social networks (9). A node’s role depends on its centrality, tie strength, and intermediary position (10). At the same time, Provan and Kenis (11) emphasized the importance of core actors, member dependence, and governance arrangements in shaping the performance of public service networks. Taket and Ann (12) likewise argued that the allocation of health and care resources for older adults requires coordination among limited supply, diverse needs, and regional equity.

These three perspectives are complementary rather than separate. Spatial justice provides the normative basis for assessing whether older adults in different areas have equitable access to services; supply–demand matching provides the substantive basis for identifying differences in service capacity across districts; and network governance moves beyond the assumption that districts operate as isolated spatial units by focusing on interdependence, tie strength, and structural position. Together, they form a logic of “equity goals-service capacity-relational structure.” Within this framework, social network analysis serves as an empirical tool for identifying relational structure. Building on measurements of district-level service capacity and transport resistance, it organizes the estimated interdistrict linkages into an analyzable network. It uses network density, centrality, and core-periphery structure to identify each district’s position within the wider pension care system. The study therefore follows a progressive analytical sequence of “identifying regional service capacity-measuring potential interdistrict linkages-analyzing overall network structure.”

### 2.2 Main developments in spatial research on pension care services

Existing research on the spatial organization of pension care services has focused first on service accessibility and has gradually moved beyond static geographic distance toward more realistic transport and behavioral conditions. Recent studies have incorporated real-time navigation data and facility preferences into an accessibility model (13). Luan et al. (14) examined accessibility from the perspective of perceived convenience and identified possible gaps between objective distance and subjective experience. The study incorporated walking, public transport, motorized travel, and facility hierarchy into a multimodal accessibility framework (15). These studies have refined the measurement of pension care accessibility. Research on resource allocation has further examined efficiency, equity, and supply–demand coordination. Research on resource allocation has combined efficiency assessment with spatial evolution to examine the allocation of institutional pension care resources (16). At the same time, improved potential models have been used to identify spatial differences in facility accessibility (17). Other studies have examined temporal changes in the accessibility and equity of pension care institutions (18) and incorporated social stratification, differentiated travel costs, and resource optimization into spatial analysis (19–21). Transport-related research has also moved from simple distance measures toward the operating conditions of public transport systems. Existing evidence shows that stop distance, waiting time, transfers, barrier-free conditions, and service reliability can all influence older adults’ access to services (22, 23). Social network analysis has subsequently been introduced to examine participation structures and relational pathways in health services for older adults. Centrality and network structure can help explain service diffusion and coordination (24).

The scope of research has also expanded from institutional care to home- and community-based care, medical support, family caregiving, and differentiated service needs. Pension care systems are shaped by institutional provision, the care workforce, urban–rural disparities, and digital reform (24). Income, living arrangements, and community relations significantly affect demand for home- and community-based care (24). Yang et al. (25) further highlighted the interdependence of medical care, nursing care, and family support. Studies of heterogeneity among older adults have also shown that needs related to daily living, emotional support, and fall prevention vary considerably (26). At the same time, family financial support and social participation can also affect patterns of care demand (27). Research on community-based integrated care (28) and long-term care utilization (29) further indicates that individual characteristics, family support, and institutional capacity jointly shape pension care needs.

### 2.3 Research gaps and analytical framework

Existing studies have provided substantial insight into the distribution of pension care facilities, spatial accessibility, resource efficiency, and supply–demand matching. Less attention, however, has been paid to potential relationships across administrative districts and to the structure of the resulting urban network. Three gaps remain. First, most studies still treat facilities, communities, or administrative districts as relatively independent spatial units. Such approaches can identify where resources are concentrated and how regions differ, but they reveal less about the potential linkages that may form between districts. Second, transport conditions are usually treated as factors affecting residents’ ability to reach services, while their role as interdistrict spatial resistance shaping the strength of cross-district linkages has received less attention. Third, even where cross-district relationships can be identified, few studies examine how these relationships are organized into an overall urban network. Core nodes, bridging positions, and core-periphery differentiation therefore remain insufficiently understood at the district level. Addressing these gaps is important because, in megacities, unequal access to pension care may arise not only from uneven local resource endowments but also from differences in districts’ ability to connect with and make use of resources elsewhere in the urban system.

This study uses Guangzhou’s 11 administrative districts as units of analysis and focuses on potential spatial linkages shaped by the joint influence of district-level service capacity and transport resistance. The analysis follows three connected levels: regional differences in pension care capacity, potential interdistrict linkages, and overall network structure. First, we construct an evaluation system covering institutional care capacity, home- and community-based care capacity, and medical support capacity for pension care, and use the entropy weighting method to measure the overall level of pension care services in each district. Second, public transport travel time characterizes interdistrict spatial resistance, and a modified gravity model estimates the strength of potential pension care linkages between districts. Social network analysis then identifies network density, centrality, and core-periphery structure, revealing districts’ structural positions and functional differences within Guangzhou’s pension care system.

## 3 Research methods

This study adopts a sequential rather than parallel methodological design. The entropy weighting method is first used to integrate pension care service indicators and measure the overall service capacity of Guangzhou’s 11 administrative districts. Min-Max normalization is then applied to standardize the scale of the composite scores and does not serve as an independent empirical analytical method. The interdistrict transport impedance index characterizes spatial travel constraints between districts. On this basis, the modified gravity model combines district-level service capacity with transport impedance to estimate the strength of potential interdistrict pension care linkages. The resulting interdistrict linkage matrix is subsequently used as relational input for social network analysis, through which network density, centrality, and core-periphery structure are examined to identify the overall network characteristics and the structural positions of individual districts. Accordingly, the methods correspond to four sequential analytical stages—node capacity measurement, spatial impedance characterization, potential linkage estimation, and network structure identification—rather than repeatedly measuring the same variable.

### 3.1 Method for analyzing the strength of spatial connections in pension care services

#### 3.1.1 Gravity model

The strength of spatial linkages in pension care services serves as a crucial foundation for identifying the coverage areas of pension care resources in megacities, as well as inter-district interdependencies and network structures. Linkage strength between regions is positively correlated with node size or attractiveness and negatively correlated with spatial resistance; this has been widely applied in research on population mobility, transportation linkages, public services, and regional networks (30). Geographical gravity models typically use a power-law function to characterize distance decay, meaning that an increase in spatial resistance reduces the likelihood of linkages forming between nodes (31). Traditional gravity models often use geographic distance as a measure of spatial impedance; however, cross-district connections for pension care services are also influenced by public transportation travel time, transfer costs, route connectivity, and age-friendly travel conditions. Relevant studies indicate that older adults’ access to public services is not determined solely by straight-line distance; public transportation travel time, distance to stops, waiting time, and the number of transfers all significantly alter their opportunities to access these services (22, 23). Therefore, drawing on the aforementioned literature, this study constructs a travel time resistance index based on inter-district public transportation travel times to modify the traditional gravity model. The model is as Equation 1:


$$I_{\mathrm{ij}}=G\frac{M_{i}M_{j}}{D_{\mathrm{ij}}^{b}}$$ (1)

The comprehensive pension care service scores derived from the entropy-weighting procedure are used as district-level node-capacity measures in the gravity model. Their role here differs from that in the preceding evaluation: the entropy-weighted scores characterize the internal service capacity of individual districts, whereas the gravity model combines these node attributes with interdistrict transport impedance to estimate potential relational strength between districts. $I_{\mathrm{ij}}$ represents the strength of the link between regions *i* and *j* in terms of eldercare services, $M_{i}$ represents the comprehensive regional pension care service development index for region *i*, $M_{j}$ represents the comprehensive regional pension care service development index for region *j*, $M_{i}M_{j}$ represents the score for the overall level of development of pension care services, $D_{\mathrm{ij}}$ represents the index of accessibility for pension transportation between region *i* and region *j,* Following previous applications of gravity models in spatial interaction and public service research (32, 33), the distance-friction coefficient *b* is set to 2. This specification assumes a quadratic distance-decay effect, meaning that the strength of potential interdistrict linkages decreases more rapidly as transport impedance increases. Because pension care services involve older adults, for whom longer travel times and transfer costs may impose relatively strong mobility constraints, a coefficient of 2 reflects a pronounced impedance effect. The gravitational constant *G* is set to 1 because it serves only as a proportional scaling parameter and does not affect the relative ranking of interdistrict linkage strengths.

#### 3.1.2 Entropy-weighted method

The entropy-weighted method objectively assigns values to indicators, avoiding the randomness associated with subjective weighting. This method requires establishing an indicator matrix; the greater the dispersion of data within the matrix, the more information it provides; the lower the information entropy, the greater the indicator’s weight; and the greater its impact on the comprehensive evaluation. Conversely, the lower the data dispersion, the less information it provides; the higher the information entropy, the lower the indicator’s weight, and the smaller its impact on the comprehensive evaluation.

Calculation steps:

(1) Data Standardization $A={\left[a_{\mathrm{ij}}\right]}_{n\times m}$


$$X_{\mathrm{ij}}=\frac{a_{\mathrm{ij}}-\min\{a_{\mathrm{ij}}\}}{\max\{a_{\mathrm{ij}}\}-\min\{a_{\mathrm{ij}}\}}$$ (2)

(2) Calculate the weight $p_{\mathrm{ij}}$ of the *j-th* indicator in the *i-th* year, that is:


$$p_{\mathrm{ij}}=\frac{X_{\mathrm{ij}}}{\sum_{i=1}^{m}X_{\mathrm{ij}}}$$ (3)

(3) Calculate the entropy value $e_{j}$ of the j-th indicator using the following formula:


$$e_{j}=-\frac{1}{\mathrm{lnm}}\sum_{i=1}^{m}(p_{\mathrm{ij}}\ln p_{\mathrm{ij}}),e_{j}=[0,1]$$ (4)

(4) Calculate the coefficient of variation $g{,}_{i}$ ，using the following formula:


$$g_{i}=1-e_{j}$$ (5)

(5) Calculate the weight $w_{j}$ of the j-th indicator, using the following formula:


$$w_{j}=\frac{g_{i}}{\sum_{j=1}^{m}g_{i}}$$ (6)

Calculate the composite score $D_{i}$ for each sample, calculating the Comprehensive Pension Care Development Index for Guangzhou’s 11 Districts Using the Entropy Method

(6) i represents the comprehensive pension care development index for Guangzhou’s 11 districts.


$$D_{i}=\sum_{j=1}^{m}w_{j}*p_{\mathrm{ij}},i=1,2,\ldots ,n$$ (7)

#### 3.1.3 Min-max data normalization method

The entropy-weighting method was used to calculate the raw scores for the comprehensive assessment of pension care service levels in Guangzhou’s 11 districts. To facilitate comparisons across districts, the composite scores were further normalized using the Min-Max normalization method. In this study, we linearly transformed the scores to the interval [0,10]. The [0,10] scale was selected to provide a more intuitive numerical representation for subsequent comparison. Because all districts are transformed using the same linear rule, the normalization preserves their relative ordering. However, the absolute magnitude of the subsequent gravity-model estimates depends on the adopted scale. The normalization formula is shown in Equation 8:


$$V^{\prime }=\frac{\left(\mathrm{new}\;\text{maxA}-\mathrm{new}\;\min\;A\right)}{\left(\text{maxA}-\text{minA}\right)}*(V-\text{minA})+1$$ (8)

Among them, V′ represents the normalized data, V represents the raw data, maxA and minA represent the maximum and minimum values of the original data column, respectively. newmaxA andnewminA represent the maximum and minimum values of the data transformation normalization interval, respectively.

#### 3.1.4 Interdistrict transport impedance index model

To characterize spatial impedance between districts, this study constructs an interdistrict transport impedance index based on public transportation travel time (18). The index represents the relative travel-time cost of interdistrict movement within Guangzhou’s public transport network. A larger value indicates greater transport impedance and, correspondingly, weaker accessibility between districts. The index is calculated as shown in Equation 9.


$$D_{\mathrm{ij}}=\frac{1}{2}[\frac{T_{\mathrm{ij}}}{\sum T_{i}/n}+\frac{T_{\mathrm{ij}}}{\sum T_{j}/n}]$$ (9)

$D_{\mathrm{ij}}$ represents the Transport impedance index between area *i* and area *j*, A larger $D_{\mathrm{ij}}$ value indicates greater travel-time impedance and weaker interdistrict accessibility $T_{\mathrm{ij}}$ represents the shortest travel time between region *i* and region *j*, $\sum T_{i}/n$ , $\sum T_{j}/n$ represents the average travel time in the shortest-distance routes between regions *i* and *j* and the other n regions.

In summary, this paper will calculate the strength of spatial linkages in pension care services across Guangzhou’s 11 districts based on the Regional Comprehensive Pension Care Development Index’s intrinsic variables( $M_{i}$ ), and inter-regional spatial linkage variables ( $D_{\mathrm{ij}}$ ), and use these findings as the basis for analyzing the spatial linkage patterns of pension care services in Guangzhou’s 11 districts.

### 3.2 Social network analysis methods

The interdistrict linkage matrix estimated by the modified gravity model serves as the relational input for the subsequent social network analysis. Social network analysis is therefore not an independent parallel estimation of pension care capacity, but a structural analysis of the potential interdistrict relationships derived in the preceding step. It is used to examine the overall network structure, node positions, and structural differentiation of pension care services in Guangzhou. By taking districts and their interrelationships as the basic units of analysis, social network analysis moves beyond treating each administrative district as an isolated spatial unit and allows the study to identify patterns of connectivity, intermediary positions, and regional dependence within older adults care service network. Borgatti et al. (10) noted that the role of a node depends not only on its own resource endowment but also on its number of direct connections, path position, and mediating capacity. Provan and Kenis (11) further showed that the performance of public service networks is closely associated with core actors, interorganizational dependence, and the overall governance structure. This method has also been applied to the study of pension health service participation and pension social relations (14).

#### 3.2.1 Network density analysis

The network density mainly measures the density of inter-regional pension connections. The greater the density, the closer the connection. The calculation formula is shown in Equation 10.


$$D=\sum_{i=1}^{k}\sum_{j=1}^{k}d(n_{i},n_{j})/k(k-1)$$ (10)

*D* is the network density, *K* is the number of node regions, and the greater the value of D, the more connections between nodes. When *d = 1*, there is a connection between network nodes, and when *d = 0*, there is no connection between nodes.

#### 3.2.2 Network centrality analysis

Network centrality analysis is a quantitative analysis of the subject’s power in the network from the perspective of relationships. It mainly includes three types of analysis: degree centrality, closeness centrality, and betweenness centrality. The specific calculation formula is shown in Equations 11–13.

##### 3.2.2.1 Centrality of point degree

Degree centrality measures the number of nodes directly connected to a pension service node in the network, reflecting the node’s ability to communicate closely with other nodes in the network and the degree of participation in the network relationship.


$$C_{\mathrm{Di}}=\sum_{i=1}^{I}r_{\mathrm{ij}}$$ (11)

$C_{\mathrm{Di}}$ stands for degree centrality, *I* represents the number of distance for degree centrality, I represents the number of other nodes associated with node *i*; $r_{\mathrm{ij}}$ representsbetweenness centrality measures the number of times a node in the network is on the shortcut distance between other nodes, reflecting the node’s control ability over other nodes and the degree of being “in the middle” of older adults’ care service transmission path of other nodes.


$$C_{\mathrm{Bi}}=\sum_{i}^{I}\sum_{K}^{I}g_{\mathrm{jk}}(i)/g_{\mathrm{jk}}$$ (12)

i≠j≠k and j<k , $C_{\mathrm{Bi}}$ stands for betweenness centrality; $g_{\mathrm{jk}}$ represents the number of shortcuts between *J* and *K*. $g_{\mathrm{jk}}$ *(i)Q* represents the number of shortcuts between *J* and *K* that must pass through node *i* to connect.

##### 3.2.2.2 Closeness centrality

Closeness centrality measures the shortcut distance between a node and other nodes (the number of lines included in the shortcut), reflecting the closeness of the older adults node to other pension nodes and the degree to which others do not control it in the network.


$$C_{\mathrm{Ci}}=1/\sum_{j=1}^{I}d_{\mathrm{ij}}$$ (13)

$C_{\mathrm{Ci}}$ stands for closeness centrality. $d_{\mathrm{ij}}$ stands for the shortcut distance between two points *i* and *j*.

#### 3.2.3 Core-periphery structure analysis

The analysis of the core-periphery structure distinguishes between a series of actors with high density, namely, the core area of pension services, and a series of actors with low density, namely, the marginal area of pension services. The exchange relationship between the core area and the marginal area is in an advantageous position, while the marginal area is in a disadvantageous position.

## 4 Index system construction and data source

### 4.1 Construction of index system

Following the principles of integrity, authenticity, reliability, and comparability, and considering the functional composition and data availability of the pension care service system in megacities, this study constructs an evaluation index system for Guangzhou from three analytical dimensions: institutional care capacity, home- and community-based care capacity, and medical support capacity for pension care. These three dimensions are not intended to represent mutually exclusive care models. Rather, they distinguish different functional components of the pension care service system according to service setting and support function. Existing studies have shown that the allocation of pension care resources cannot be assessed solely by the number of institutions or beds, but should also consider community-based services, medical resources, and professional support in order to reflect the functional complementarity among different components of the care system (8, 34).

Institutional care capacity reflects the scale and carrying capacity of residential and centralized pension care services in each district. Li et al. (16) pointed out that the number of pension care institutions, service investment, and resource utilization efficiency jointly affect the service level of pension care institutions. Li et al. (17) further found that the spatial equity of institutional pension care facilities is closely related to facility capacity, population demand, and location conditions. Accordingly, this study selects the number of pension care institutions per 1,000 older adults, the number of pension care beds per 1,000 older adults, the number of municipal-level comprehensive pension care institutions, and the number of district-level comprehensive pension care institutions to reflect institutional coverage, bed capacity, and the allocation of centralized care resources. Although some institutional care facilities may provide basic medical or nursing services internally, such services are treated here as embedded components of institutional care when they are delivered within the organizational setting of the pension care institution.

Home- and community-based care capacity reflects the extent to which older adults can obtain daily assistance, care, rehabilitation, and other forms of support while remaining in familiar home and community settings. Li et al. (35) found that living conditions, neighborhood relations, and community mutual-aid organizations significantly affect older adults’ demand for home- and community-based pension care services. Accordingly, this study selects the number of community dining facilities per 1,000 older adults, the number of family nursing bed service institutions, the number of comprehensive pension care institutions in streets and towns, and the number of community health service stations to characterize service convenience, grassroots coverage, and the continuity of community-based care.

Medical support capacity for pension care reflects the broader district-level medical resources available to support older adults’ diagnosis, treatment, rehabilitation, and nursing needs. Unlike medical services embedded within individual pension care institutions, this dimension captures the external medical support environment available across the district. Liu et al. (1) pointed out that the type, level, and supply–demand relationship of medical and care facilities should be considered simultaneously. Recent studies have further incorporated perceived convenience into accessibility assessment, revealing possible discrepancies between objective distance and subjective experience (14, 36). Accordingly, this study selects the number of medical institutions, medical beds, and health professionals per 1,000 older adults to reflect the coverage, service capacity, and professional support of the medical system for pension care. This distinction helps avoid equating medical services provided within individual pension care institutions with the broader district-level medical support capacity available to pension care services.

To reduce the influence of differences in the size of the older population across districts, all indicators are standardized per 1,000 older adults. This treatment improves comparability across districts and allows the evaluation results to more accurately reflect the allocation of pension care resources relative to the size of the older population. Table 1 presents the specific indicators.

**Table 1:** Evaluation index of pension care service level.

Target layer	Criterion layer	Index layer	Unit	Code
Pension service supply level	Institutional care capacity	Number of pension care institutions per thousand	Quantity/thousand people	X11
		Number of pension care beds per thousand	Quantity/thousand people	X12
		Municipal comprehensive pension institutions per thousand people	Quantity/thousand people	X13
		District-level comprehensive pension institutions per 1,000 people	Quantity/thousand people	X14
	Home- and community-based care capacity	Number of dining rooms for older adults per thousand	Quantity/thousand people	X21
		Number of family nursing bed service institutions for the oldsters per thousand	Quantity/thousand people	X22
		Number of comprehensive pension institutions in streets and towns per thousand oldsters	Quantity/thousand people	X23
		Number of community health service stations per thousand oldsters	Quantity/thousand people	X24
	Medical support capacity for pension care	Number of medical institutions per thousand oldsters	Quantity/thousand people	X31
		Number of medical beds per thousand oldsters	Quantity/thousand people	X32
		Number of health technicians per thousand oldsters	Quantity/thousand people	X33

### 4.2 Data sources

Data on pension care service indicators were drawn mainly from the Guangzhou Statistical Yearbook 2024 and publicly available statistical information on pension care services released by the Guangzhou Municipal Civil Affairs Bureau. Indicators relating to institutional care, home- and community-based care, and medical support capacity for pension care were compiled from statistics on pension care institutions, pension care beds, community-based care facilities, medical institutions, hospital beds, and health professionals reported in the Guangzhou Statistical Yearbook. Data on the older population were used to standardize the relevant indicators as the number per 1,000 older residents, thereby improving comparability across districts. Interdistrict public transportation travel times were obtained from Gaode Map. The locations of the 11 district governments in Guangzhou were used as representative district-level points, and the shortest travel times under a combined mode of bus, metro, and walking were recorded under consistent travel-mode and query conditions. These travel-time data were then used to construct the interdistrict transport impedance index.

## 5 An empirical study on the spatial connection of pension care services in Guangzhou

### 5.1 Analysis of the development level of pension care services in Guangzhou

This paper calculates the development level of pension care services in Guangzhou’s 11 districts from three dimensions: institutional care capacity, home- and community-based care capacity, and medical support capacity, using Equations 2–8. As shown in Table 2, comprehensive pension care service levels differ significantly across districts. The highest value is 0.9772 in Baiyun District, and the lowest value is 0.0774 in Haizhu District, with a range of 0.8998, reflecting the strong spatial imbalance in older adults’ care resource allocation and service coordination ability.

**Table 2:** Pension service level.

Region	Institutional care capacity	Home- and community-based care capacity	Medical support capacity for pension care	Comprehensive pension service level	Standard value	Sort
Baiyun	0.8926	0.6158	0.8944	0.9772	10.000	1
Tianhe	0.1157	0.4786	0.8972	0.5618	5.845	2
Liwan	0.7059	0.2580	0.0526	0.4353	4.580	3
Nansha	0.2150	0.6677	0.1182	0.4183	4.410	4
Huangpu	0.1445	0.2004	0.5166	0.3595	3.822	5
Panyu	0.1499	0.4309	0.3106	0.3495	3.721	6
Conghua	0.1423	0.6197	0.1487	0.3324	3.551	7
Huadu	0.1802	0.2569	0.2966	0.2480	2.707	8
Zengcheng	0.0683	0.3170	0.2713	0.1960	2.186	9
Yuexiu	0.0146	0.0609	0.4759	0.1600	1.826	10
Haizhu	0.0568	0.0678	0.1640	0.0774	1.000	11

Baiyun District ranks first in terms of comprehensive pension care service level. The level of institutional pension care service and the medical support capacity for pension care are close to 0.90. Community pension care service is also strong. This forms a comprehensive structure for coordinated development of institutional care, community support, and medical resources, and has a strong ability to carry out and expand pension care services. Tianhe District’s comprehensive index is 0.5618, ranking second, and its medical support capacity for pension care and pension is 0.8972, the highest in the city; however, its institutional pension service level is relatively low, reflecting a medical-resource-driven structure. Liwan District’s comprehensive index is 0.4353, and the service level of institutional pension care is 0.7059. The advantages mainly come from the allocation of institutions and beds, but the medical support capacity for pension care is relatively insufficient.

The community pension care service levels in Nansha District and Conghua District are 0.6677 and 0.6197, respectively. Relying on older adults’ canteens, community health service stations, and family pension care service institutions, the district has built a strong grassroots service network, but institutional care and medical support capacity remain insufficient. The medical support capacity for pension care in Huangpu District is 0.5166, and the three dimensions in Panyu District are relatively balanced. Still, the service level of institutional pension is 0.1499, the service level of community pension is 0.4309, and the medical support capacity for pension care is 0.3106. Huadu District and Zengcheng District are low- and medium-level overall and have not yet developed outstanding service advantages. Although Yuexiu District has a high level of medical support capacity for pension care, institutional and community pension resources are clearly insufficient relative to the size of the older adult population. Haizhu District is low in all three dimensions, and the gap between pension service supply and the demand of an aging population is more pronounced.

Overall, the development of pension care services in Guangzhou shows a multi-center, functionally differentiated spatial pattern. Baiyun District is a comprehensive supply center. Tianhe District and Huangpu District have the medical support capacity for pension care. Liwan District excels at institutional care for the aged, while community pension care services mainly support Nansha District and Conghua District. These differences in resource endowments across regions provide a realistic basis for constructing a cross-regional pension service network and promoting collaborative resource allocation.

### 5.2 Analysis of interdistrict transport impedance in Guangzhou

Taking the 11 district governments as representative district-level nodes, this study uses Gaode Map to obtain the shortest public transportation travel time under a combined mode of bus, metro, and walking. It calculates the interdistrict transport impedance index accordingly. A lower impedance value indicates a lower travel-time cost and stronger interdistrict accessibility, whereas a higher value indicates greater spatial impedance. Table 3 reports the results. Guangzhou’s traffic accessibility shows a relatively clear center-periphery gradient. Tianhe District has an average public transport time of 65.9 min and a transport impedance index of 8.1633, the lowest among the 11 districts, indicating that public transport connections between Tianhe District and other regions are the most convenient. The travel time between Tianhe District and Haizhu District, Baiyun District, Huangpu District, and Panyu District is relatively short, which has the regional advantage of connecting the central urban area, eastern urban area, and southern urban area, and may assume a strong intermediary and referral function in the cross-regional allocation of pension service resources.

**Table 3:** Interdistrict transport impedance index and connectivity ranking of Guangzhou’s districts.

Region	Average public transport time (minutes)	Transport impedance index	Sort
Tianhe	65.9	8.1633	1
Haizhu	77.7	8.7768	2
Baiyun	78.6	8.8595	3
Yuexiu	79.2	8.8846	4
Panyu	82.0	9.0526	5
Liwan	86.0	9.2134	6
Huangpu	88.3	9.5968	7
Nansha	110.2	10.8163	8
Huadu	118.0	11.2901	9
Conghua	136.6	12.5646	10
Zengcheng	141	12.837	11

The relatively low impedance values of Haizhu District, Baiyun District, and Yuexiu District are 8.7768, 8.8595, and 8.8846, respectively, indicating comparatively high accessibility. Haizhu District is convenient for connecting Tianhe District, Panyu District, and Nansha District, and has the spatial conditions to connect the central urban area and southern Guangzhou; Baiyun District has the function of connecting the central urban area and the northern region; Although pension care institutions and community pension resources in Yuexiu District are relatively limited, its traffic location is conducive to the transmission of medical resources and professional pension services to other regions.

The transport impedance indices of Panyu District, Liwan District, and Huangpu District range from 9.0526 to 9.5968, placing them at a medium level within the city. Panyu District is relatively well connected with Haizhu District, Tianhe District, and Nansha District and may therefore serve as an important node in the southern pension care service network. Liwan District has convenient connections with Yuexiu District, Baiyun District, and other central districts, which may help extend the advantages of its institutional care capacity. Huangpu District also has relatively favorable connections with Tianhe District and Zengcheng District, giving it the potential to link the central urban area with the eastern peripheral area.

The Transport impedance index of Nansha District, Huadu District, Conghua District, and Zengcheng District is relatively high. Among them, Conghua District and Zengcheng District reached 12.5646 and 12.8369, respectively. The time cost of public transport was high, reflecting the limited convenience of accessing the city’s pension service network. Low traffic access may limit the cross-regional spread of pension service resources, older adults’ access to professional care across districts, and family members’ visits. In sum, Guangzhou’s transportation network has a spatial structure with Tianhe, Haizhu, Baiyun, and Yuexiu as high-access nodes and gradually decaying in the southern, northern, and eastern peripheral urban areas, providing a distance-based basis for calculating the spatial connection strength of subsequent pension services.

### 5.3 Spatial network connection strength of pension care services in Guangzhou

Based on the results in Tables 2, 3, and using the modified gravity model, we construct the spatial network connection model of pension care services in Guangzhou. Based on the development level of comprehensive pension care services in each district and the interdistrict transport impedance index, we calculate the spatial connection strength of pension care services among Guangzhou’s 11 districts. By summing the 10 interregional contact intensities for each district as the contact sender, we obtain the total spatial network connection strength of pension services in each district, as shown in Table 4.

**Table 4:** Total strength of spatial network connections of pension care services in Guangzhou.

Region	Total spatial network connections	Proportion	Sort	Cumulative percentage
Baiyun	390.632	21.700%	1	21.700%
Tianhe	260.599	14.476%	2	36.176%
Liwan	202.168	11.231%	3	47.407%
Nansha	170.719	9.484%	4	56.890%
Panyu	162.612	9.033%	5	65.923%
Huangpu	158.428	8.801%	6	74.724%
Conghua	130.092	7.227%	7	81.951%
Huadu	106.211	5.900%	8	87.851%
Yuexiu	89.693	4.983%	9	92.833%
Zengcheng	80.449	4.469%	10	97.302%
Haizhu	48.560	2.698%	11	100.000%

The linkage strengths estimated by the modified gravity model represent potential interdistrict spatial relationships derived from district-level service capacity and transport impedance, rather than directly observed service flows such as cross-district admissions, medical referrals, or institutional collaborations. The results show that the spatial network connections of pension care services in Guangzhou have clear characteristics of regional concentration and hierarchical differentiation. The top five regions by total connections are Baiyun District, Tianhe District, Liwan District, Nansha District, and Panyu District, with a total of 1186.730, accounting for 65.923% of the city’s spatial network connections for pension care services. Among them, Baiyun District had 390.632 connections (21.700%), ranking first in the city; Tianhe District had 260.599 connections (14.476%). Baiyun District and Tianhe District accounted for 36.176% of the total, constituting the main central node in Guangzhou’s spatial network of pension care services. The total number of connections between Nansha District and Panyu District was 170.719 and 162.612, respectively, accounting for 18.517% of the city total. Nansha District has a high level of community pension care service and forms a strong connection with the central urban area through Panyu District; Panyu District is relatively balanced in the three dimensions of institutional pension, community pension, and the medical support capacity for pension care. At the same time, it has the location conditions to connect Haizhu District, Tianhe District, and Nansha District, so it has become an important node in the older adults service network in southern Guangzhou. The total number of connections in Huangpu District was 158.428, accounting for 8.801%, and it mainly relied on the medical-nursing combination level and its connections with Tianhe District and Baiyun District to form a strong network position.

The spatial connection of pension services in Guangzhou is mainly concentrated between Baiyun District, Tianhe District, Liwan District, and other core nodes. The strongest connection is between Baiyun District and Tianhe District, with a connection strength of 81.366; Tianhe District to Baiyun District is 79.252; Liwan District to Baiyun District is 63.295; and Baiyun District to Liwan District is 60.542. The connection strength between Nansha District and Baiyun District reached 41.825 and 41.028, respectively, indicating that Baiyun District has strong potential for cross-district resource connectivity between central and peripheral urban areas.

A total of 110 groups of directional pension care service spatial connections were formed in 11 districts of Guangzhou, of which 72 groups’ connection intensity was 16.365 lower than the average value of the whole city, accounting for 65.455% of the total connection portfolio; The contact strength of 48 groups was lower than 10, accounting for 43.636%. This shows that although pension care services in Guangzhou have formed a network structure with Baiyun District and Tianhe District as the core, most interregional connections remain relatively weak. Yuexiu District, Zengcheng District, and Haizhu District accounted for less than 5% of total connections, with Haizhu District’s total connections at only 48.560 (2.698%). Pension service resources differ markedly in connection strength across regions, and there is still room to improve cross-regional resource sharing, medical pension referral, and community pension coordination.

### 5.4 Spatial connection of pension care services in 11 districts of Guangzhou

#### 5.4.1 Establishment of network model

Based on the spatial connection strength of pension care services among 11 districts in Guangzhou, and treating each district as a network node, we used social network analysis to construct the spatial connection network of pension care services. The original contact strength matrix was obtained using UCINET6 software. After removing self-connections, we identified 110 directed interregional connections among the 11 areas, as shown in Figure 1.

**Figure 1:**
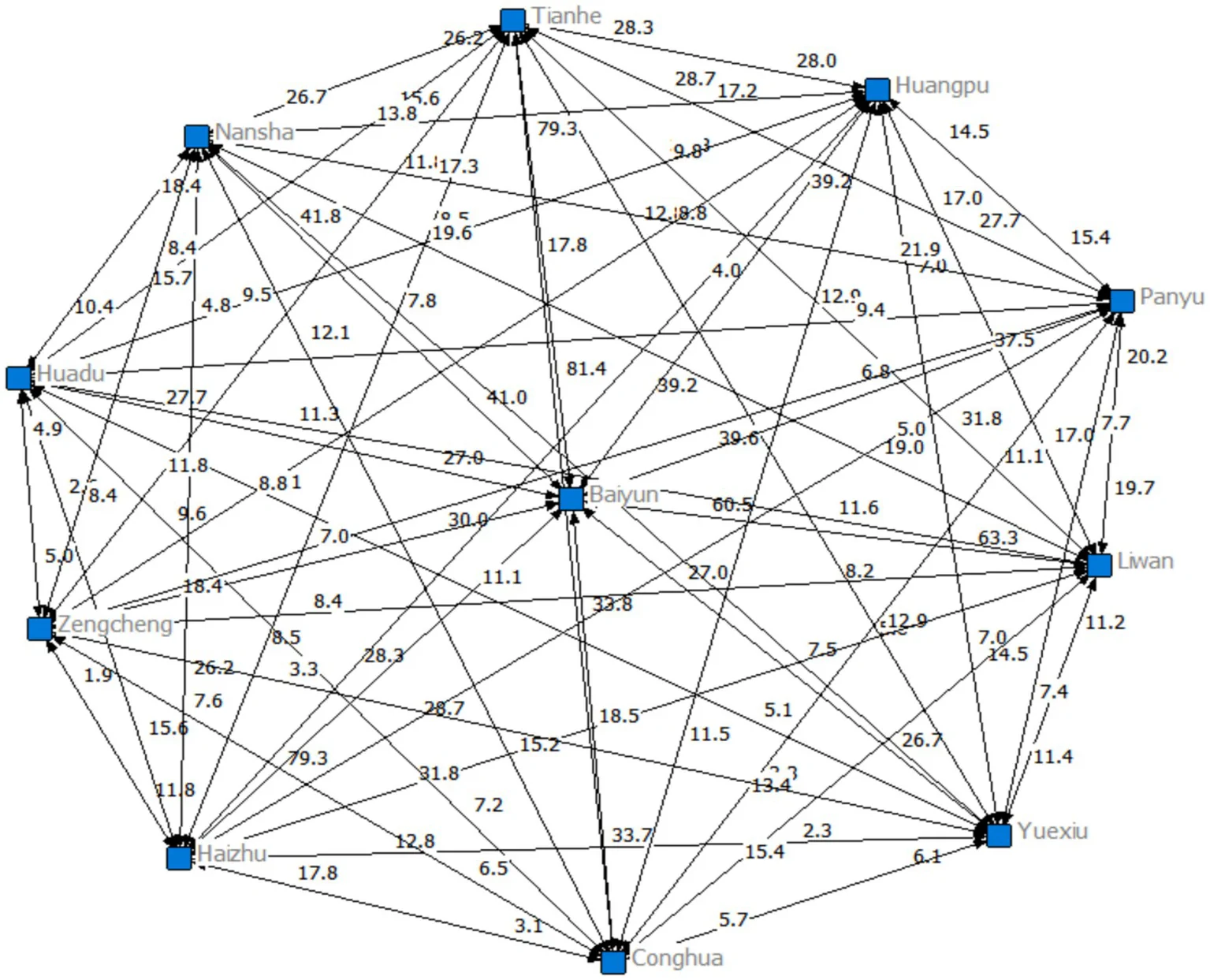
Structure chart of spatial connection network of senile care services in 11 districts of Guangzhou.

Because using the median link strength as the breakpoint would isolate Haizhu District and affect overall network connectivity, this paper sets a link-strength threshold of 10 as the binary breakpoint, based on link recognition and network integrity. If the connection strength is greater than 10, the value is 1, indicating a relatively strong spatial connection between the two regions; if the connection strength does not exceed 10, the value is 0, and the diagonal of the matrix is set to 0. Table 5 shows the binarized results.

**Table 5:** Spatial connection dichotomy matrix of pension care services in Guangzhou.

City	Liwan	Yuexiu	Haizhu	Tianhe	Baiyun	Huangpu	Panyu	Huadu	Nansha	Conghua	Zengcheng
Liwan	0	1	0	1	1	1	1	1	1	1	0
Yuexiu	1	0	0	1	1	0	0	0	0	0	0
Haizhu	0	0	0	0	1	0	0	0	0	0	0
Tianhe	1	1	0	0	1	1	1	1	1	1	1
Baiyun	1	1	1	1	0	1	1	1	1	1	1
Huangpu	1	0	0	1	1	0	1	0	1	1	0
Panyu	1	0	0	1	1	1	0	0	1	1	0
Huadu	1	0	0	1	1	0	0	0	1	0	0
Nansha	1	0	0	1	1	1	1	1	0	1	0
Conghua	1	0	0	1	1	1	1	0	1	0	0
Zengcheng	0	0	0	1	1	0	0	0	0	0	0

Among the 110 possible interregional relationships, 61 connections exceeded the breakpoint value, accounting for 55.45% of the theoretical maximum number of connections. The dichotomy matrix was imported into UCINET, and NetDraw was used to draw the structure diagram of the spatial connection network of older adults’ care services in 11 districts of Guangzhou, as shown in Figure 2.

**Figure 2:**
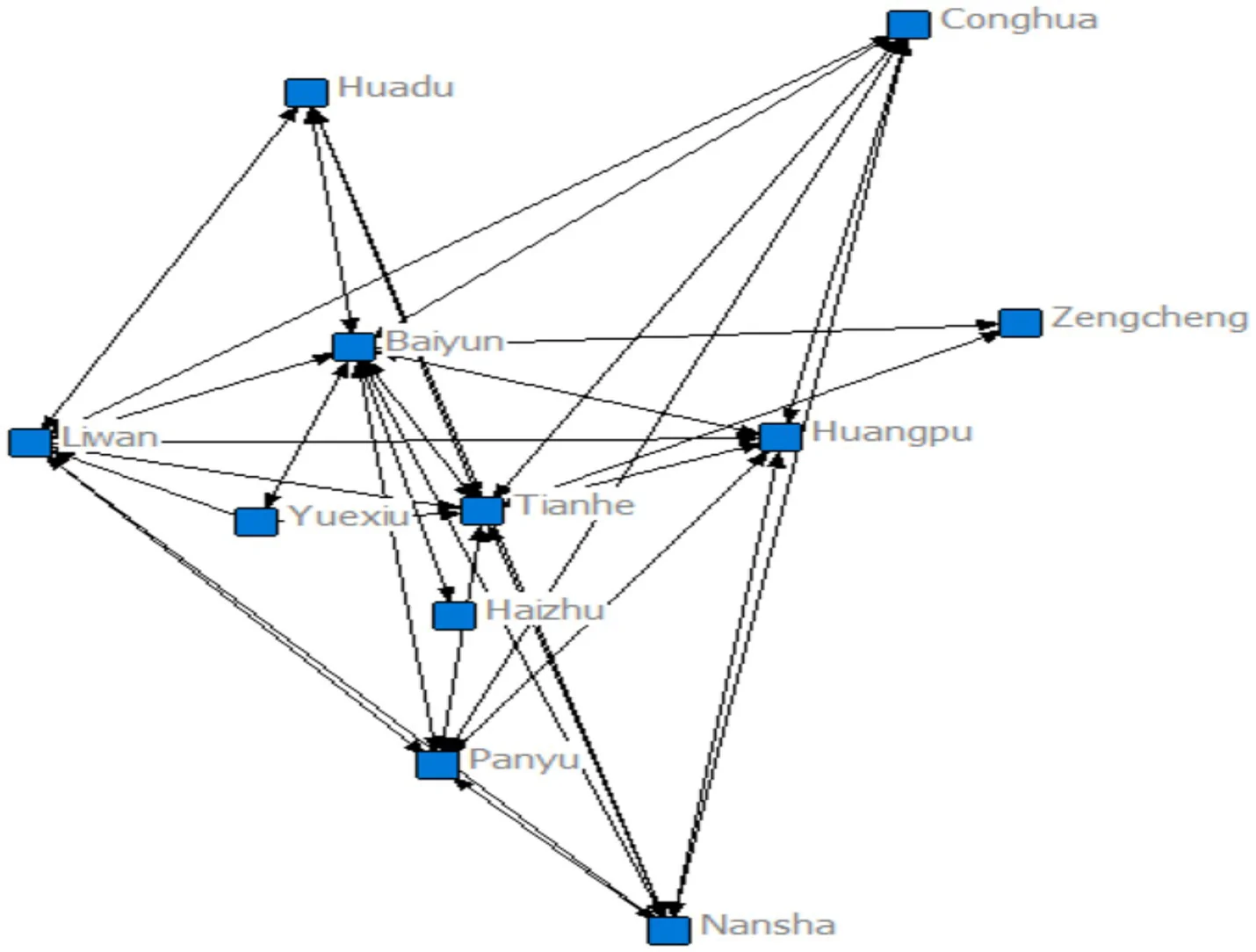
Structure chart of binary spatial connection network of pension services in 11 districts of Guangzhou.

#### 5.4.2 Analysis of the characteristics of the network connection structure of pension care services in Guangzhou

##### 5.4.2.1 Network density analysis

Network density is calculated as the proportion of observed binary ties in the estimated network relative to the theoretical maximum number of possible ties. According to Table 5 and Equation 10, we calculate the network density of the Guangzhou pension care service network. The theoretical maximum number of connections among 11 nodes is 121; the actual number of connections in the binary network matrix is 61; and the overall network density is 0.5545, indicating that potential spatial linkages among districts are relatively dense. However, the intensity of network participation varies across districts, and there is a certain gap in the degree of tightness of the older adults care service network in different regions.

##### 5.4.2.2 Network centrality analysis

According to Table 5 and Equations 11–13, the degree centrality, betweenness centrality, and closeness centrality of the connection structure of the older adults care service network in Guangzhou are calculated, and the results are shown in Table 6.

**Table 6:** Ranking table of contact centrality of Guangzhou pension care service network.

Item No	Degree centrality				Closeness centrality				Betweenness centrality	
	Out-degree		Indegree		Out-degree		Indegree			
1	Baiyun	10	Baiyun	10	Baiyun	100.000	Baiyun	100.000	Baiyun	31.167
2	Tianhe	9	Tianhe	9	Tianhe	90.909	Tianhe	90.909	Tianhe	13.167
3	Liwan	7	Liwan	8	Liwan	76.923	Liwan	83.333	Liwan	3.167
4	Nansha	7	Nansha	7	Nansha	76.923	Nansha	76.923	Nansha	1.500
5	Panyu	6	Panyu	6	Panyu	71.429	Panyu	71.429	Panyu	0.000
6	Huangpu	6	Huangpu	6	Huangpu	71.429	Huangpu	71.429	Huangpu	0.000
7	Conghua	6	Conghua	6	Conghua	71.429	Conghua	71.429	Conghua	0.000
8	Huadu	4	Huadu	4	Huadu	62.500	Huadu	62.500	Huadu	0.000
9	Yuexiu	3	Yuexiu	2	Yuexiu	58.824	Yuexiu	55.556	Yuexiu	0.000
10	Zengcheng	2	Zengcheng	2	Zengchengng	55.556	Zengcheng	55.556	Zengcheng	0.000
11	Haizhu	1	Haizhu	1	Haizhu	52.632	Haizhu	52.632	Haizhu	0.000

###### 5.4.2.2.1 Degree centrality

As shown in Table 6, the average out-degree and in-degree of the Guangzhou pension Care Service spatial connection network are both 5.545, and the out-degree and in-degree centrality are both 49.000%, indicating a clear concentration in the potential outward and inward connectivity of pension care services across districts. In terms of out-degree centrality, Baiyun District has an out-degree of 10, establishing strong links with the other 10 districts and serving as the most extensive area in the city’s pension service spatial network; Tianhe District ranked second with an out-degree of 9. The out-degrees of Baiyun District, Tianhe District, Liwan District, and Nansha District were 10, 9, 7, and 7, respectively, accounting for 54.10% of the total 61 external contacts, indicating that the cross-regional radiation of the city’s pension care service resources is mainly concentrated in these areas. Panyu District, Huangpu District, and Conghua District are at the middle level of the network, with an out-degree of 6; Huadu District, Yuexiu District, Zengcheng District, and Haizhu District have relatively low visibility, and their direct external contact scope is relatively limited.

In terms of in-degree centrality, Baiyun District and Tianhe District have values of 10 and 9, respectively, indicating that these two districts have strong external resource absorption and cross-regional coordination ability while radiating pension service resources outward. The point-in degree of Liwan District is 8, which is higher than the point-out degree of 7, indicating that its ability to absorb external pension service connections is slightly stronger than its potential to receive interdistrict pension care linkages is slightly stronger than its potential outward connectivity; Yuexiu District has a point-out degree of 3 and a point-in degree of 2, showing a relatively strong tendency of external contact. Nansha District, Panyu District, Huangpu District, Conghua District, Huadu District, Zengcheng District, and Haizhu District have the same point-out degree and point-in degree, and the relationship between the output and absorption of pension service resources is generally balanced. Among them, Haizhu District has a point-out degree and point-in degree of 1, and only a few core nodes connect to the city’s network, leaving it on the network’s periphery. Overall, the spatial network of pension care services in Guangzhou has formed a structure with Baiyun District as the main core, Tianhe District as the sub-core, Liwan District and Nansha District as important supporting nodes, and other regions participating hierarchically.

###### 5.4.2.2.2 Closeness centrality

Out-closeness centrality reflects the convenience of establishing pension service links between a region and other regions through direct or indirect paths. The higher the out-closeness centrality, the fewer intermediate links the region needs to pass through to transfer pension service resources to other regions; the higher the degree of access to the center, the more efficient the region is at absorbing external pension service resources and accessing the cross-regional collaborative network. As shown in Table 6, the average out-close centrality of Guangzhou’s pension care service spatial network is 71.687, and the standard deviation is 13.815; The average value of the closeness centrality is 71.972, and the standard deviation is 14.464, indicating that the overall accessibility of the city’s pension care service network is good. However, significant differences remain across regions.

Baiyun District ranks first in the city in both outgoing and incoming centrality (100), indicating that it can directly connect the remaining 10 districts and has the strongest network independence in delivering, absorbing, and coordinating pension service resources across regions. Tianhe District and Baiyun District are both close to the center, with a value of 90.909, ranking second. Together with Baiyun District, Tianhe District forms the highly accessible core of the city’s pension care service network. Liwan District’s in-closeness centrality is 83.333, higher than its out-closeness centrality of 76.923, indicating that it is relatively easy for other regions to reach Liwan District, and that its pension service resource absorption capacity is slightly stronger than its external radiation efficiency. Nansha District’s centrality is 76.923, and Panyu District, Huangpu District, and Conghua District’s centrality is 71.429, which is at the middle level of the network. Huadu District, Yuexiu District, Zengcheng District, and Haizhu District have relatively low centrality, and Haizhu District’s two indicators are both 52.632, indicating that it needs more intermediate links to establish strong ties with other regions and depends heavily on core nodes such as Baiyun District. In-closeness centrality shows that the whole network is 65.09 and 65.75%, respectively, indicating that accessibility in the older adults care service network is mainly concentrated in a few core areas.

###### 5.4.2.2.3 Betweenness centrality

Betweenness centrality measures the extent to which a region lies on the shortest paths between other regions, reflecting how much a district serves as a potential bridge between other districts within the estimated network. Higher betweenness centrality indicates stronger organizational and connection capacity in the older adults care service network in this region. As shown in Table 6, the average betweenness centrality of the Guangzhou pension care service network is 4.455, with a standard deviation of 9.238. The intermediary role varies widely across regions. Among the 11 districts, only Baiyun District, Tianhe District, Liwan District, and Nansha District have betweenness centrality greater than 0. In comparison, the remaining 7 districts do not lie on the shortest path between other nodes.

The betweenness centrality of Baiyun District is 31.167, ranking first, indicating that it is mostly on the key path of the connection of pension services in different regions, and it is the main hub for the integration of pension service resources and occupies the strongest potential bridging position in the estimated interdistrict network in the city. Tianhe District, with a betweenness centrality of 13.167, ranks second and plays an important role in connecting the central urban area, the eastern urban area, and the southern urban area. Baiyun District and Tianhe District account for 90.48% of the network’s total betweenness centrality, indicating that the city’s pension care service network is highly centralized in these two core areas. The betweenness centrality of Liwan District and Nansha District was 3.167 and 1.500, respectively, suggesting secondary intermediary functions. Although Panyu District, Huangpu District, and Conghua District have more direct connections, their betweenness centrality is 0, indicating that they mainly serve as supply nodes or demand-receiving nodes for pension care services and have not yet become irreplaceable transmission channels between other regions. The potential of the network-wide intermediary center is 32.65%, indicating clear intermediary concentration and core path dependence in the spatial connection of pension care services in Guangzhou.

###### 5.4.2.2.4 Core-periphery structure analysis

The core-periphery structure analysis is used to identify the structural position of each district in the spatial connection network of pension care services in Guangzhou, and reveal the core area, the peripheral area, and their interrelationships. The core area usually has a high network connection density and plays an important role in allocating pension service resources, transmitting information, and facilitating cross-regional referrals; the marginal area has relatively low connection density and depends to some extent on the core area.

As shown in Table 7, Liwan District, Tianhe District, and Baiyun District are core areas, while the remaining eight areas are at the periphery of the network, indicating clear structural layers in Guangzhou’s spatial network of pension care services. The internal density of the core area is 1.000, indicating that the three core areas have formed strong two-way links and strong synergy in sharing pension care facilities, allocating medical care resources, and connecting cross-regional services. The density from the core area to the peripheral area is 0.833, which reflects that the core area has a strong radiation and driving effect on older adults’ care services in the peripheral area. The density from the marginal area to the core area is 0.875, which is higher than that from the core area to the marginal area, indicating stronger structural dependence of peripheral districts on core nodes within the estimated network. The internal density of the marginal area is only 0.250, indicating that horizontal connections among peripheral areas are relatively weak, and some pension care services still rely on core nodes. In general, the spatial network of pension care services in Guangzhou presents the structural characteristics of “close internal core area, strong core peripheral interaction, and insufficient internal coordination in the peripheral area”.

**Table 7:** The core-periphery structure of the spatial network of pension care services in Guangzhou.

Core-periphery structure	Node city	Density matrix	
		Core area	Marginal zone
Core area	Liwan, Tianhe, Baiyun	1.000	0.833
Marginal zone	Yuexiu, Haizhu, Huangpu, Panyu, Huadu, Nansha, Conghua, Zengcheng	0.875	0.250

## 6 Conclusions and countermeasures

### 6.1 Research conclusion

This study reveals that the spatial organization of pension care services in Guangzhou is shaped not only by differences in district-level service capacity, but also by transport impedance and network position. The findings indicate that pension care resources in a megacity should not be understood solely as territorially bounded stocks within individual administrative districts. Rather, their potential influence depends on how districts are connected within the wider urban service network.

First, pension care services develop with clear spatial differentiation across Guangzhou. Baiyun District shows the strongest comprehensive service capacity; Tianhe and Huangpu have relative advantages in medical support capacity for pension care; Liwan is characterized by stronger institutional care capacity, while Nansha and Conghua perform relatively well in community-based care. At the same time, transport impedance generally increases from the central districts toward the peripheral districts. These findings suggest that differences in pension care provision reflect not only resource quantity but also functional specialization and uneven spatial conditions for accessing and connecting services.

Second, the estimated interdistrict network exhibits clear centralization and hierarchical differentiation. Baiyun and Tianhe occupy prominent structural positions, while Liwan, Nansha, and Panyu provide important supporting connections. However, district-level service capacity and network position are not fully equivalent. A district with relatively strong local resources does not necessarily occupy the same structural position in the wider network, because service capacity and transport impedance jointly shape potential interdistrict connectivity. This indicates that pension care provision in megacities cannot be adequately understood through administrative-unit comparisons alone.

Third, Guangzhou’s pension care service network displays a distinct core-periphery structure. Liwan, Tianhe, and Baiyun form the core, while peripheral districts show weaker horizontal connections and stronger structural dependence on core nodes. This pattern suggests that cross-district coordination may remain vulnerable when interdistrict connections are concentrated in a small number of core districts. Strengthening horizontal connections among peripheral districts is therefore important for reducing excessive structural dependence on a limited number of central nodes.

Overall, the findings extend understanding of spatial equity in urban pension care from a territorial perspective to a relational one. Spatial equity depends not only on whether resources are evenly distributed across districts, but also on whether districts can effectively connect to and use resources located elsewhere in the city. From this perspective, the spatial organization of pension care services involves both territorial resource equity and relational network accessibility. This provides a broader basis for understanding public service coordination in rapidly aging megacities.

### 6.2 Development countermeasures

First, priority should be given to establishing a coordinated service mechanism among the core districts. Baiyun District can serve as a comprehensive coordination node because of its relatively strong overall pension care capacity; Tianhe District can play a stronger role in connecting medical support capacity for pension care resources; and Liwan District can strengthen the sharing of institutional care capacity. Rather than simply expanding facilities, the priority should be to establish a standardized cross-district coordination mechanism covering bed availability, referral applications, receiving confirmation, service records, and follow-up feedback. A city-level digital platform could integrate information on available beds, medical resources, rehabilitation services, community care, and professional personnel, while designated district-level agencies would handle cross-district coordination and case transfers.

Second, differentiated sub-centers should be developed according to the functional strengths of individual districts. Panyu can be strengthened as a southern connecting node linking the central city with Nansha; Huangpu can serve as an eastern node centered on medical support capacity for pension care; Conghua can strengthen community- and home-based care functions in northern Guangzhou; and Nansha can further develop community-based service capacity in the southern part of the city. These sub-centers are not meant to replicate the functions of Baiyun or Tianhe, but to reduce excessive dependence on the core by improving regional self-sufficiency and cross-district connectivity.

Third, differentiated interventions should be adopted for districts with weaker network participation. For Haizhu and Yuexiu, where local pension care provision is relatively weak compared with the size of the older population, policy attention should focus on strengthening community-based and institutional care capacity. For Huadu, Conghua, and Zengcheng, where transport impedance is relatively high, priority should be given to improving public transport connections, age-friendly travel conditions, and service coordination with adjacent districts. At the same time, horizontal cooperation among peripheral districts should be encouraged in areas such as shared beds, community meal services, rehabilitation care, home-based nursing beds, and emergency assistance.

To improve policy implementability, the effectiveness of cross-district coordination could be monitored through a set of operational indicators, including cross-district referral response time, the proportion of institutions connected to the citywide information platform, the coverage of shared bed information, the number of cross-district collaborative service projects, and changes in network connectivity among peripheral districts. These indicators would allow Guangzhou to evaluate whether the proposed network-based coordination mechanisms are functioning as intended.

### 6.3 Research contribution

The theoretical contribution of this study lies in shifting the analytical focus from territorially bounded resource allocation toward relational public service networks, showing that the spatial equity of pension care in megacities is shaped jointly by local service capacity, transport impedance, and network position. This extends the understanding of pension care provision beyond differences in resource stocks across administrative districts and highlights the importance of interdistrict relational structure in shaping potential service accessibility and coordination.

Methodologically, this study develops an integrated analytical framework combining comprehensive evaluation, transport impedance measurement, a modified gravity model, and social network analysis. This framework links district-level service capacity with interdistrict spatial resistance and potential relational strength, making it possible to identify not only differences in local pension care provision but also the structural positions of districts within the wider urban service network.

In practical terms, the study identifies core nodes, supporting nodes, and peripheral districts within Guangzhou’s pension care service network, providing a more differentiated basis for cross-district resource coordination, sub-center development, service referral design, and the improvement of pension care provision in areas with weaker network participation.

### 6.4 Research limitations and prospects

This study reflects the cross-sectional characteristics of the spatial network of pension care services in Guangzhou, and has not yet revealed the dynamic evolution of the network structure. The modified gravity model is based on the potential connection formed by the level of pension care service and traffic conditions, and there are still differences with the actual flow of older adults, such as cross regional check-in, medical referral and institutional cooperation. The follow-up study can build multi-year data, and further analyze the evolution law and actual operation effect of the spatial network of pension care services by combining the operation records of pension care institutions, cross regional service flow and pension survey data.

## Data Availability

The original contributions presented in the study are included in the article/supplementary material, further inquiries can be directed to the corresponding author.

## References

[1] Liu Y Ye C Wu Y Fang K Mi J. Assessing spatial equity in integrated health care facilities for older adults: a case study in the metropolis of Guangzhou, China. Front Public Health. (2026) 14:1890540. doi: 10.3389/fpubh.2026.1890540, 42517108 PMC13402153

[2] Baquero Larriva MT Alonso A Higueras García E. Spatial justice and hyper-accessibility for older adults: a comparative study of Madrid and Munich. Land (Basel). (2026) 15:1141. doi: 10.3390/land15071141

[3] Marston HR van Hoof J Yon Y. Digitalising the built environment for all generations: a new paradigm for equity and inclusive age-friendly cities and communities. Indoor Built Environ. (2024) 33:213–7. doi: 10.1177/1420326X231176621

[4] Wang Z Zhou Y. Integrating health status transitions and service demands: a spatial framework for pension care service resource allocation. ISPRS Int J Geo Inf. (2026) 15:83. doi: 10.3390/IJGI15020083

[5] Zhang Y Zhang M Hu H He X. Spatio-temporal characteristics of the supply and demand coupling coordination of pension care service resources in China. Int J Environ Res Public Health. (2022) 19:10397. doi: 10.3390/ijerph191610397 PMC9408112 36012031

[6] Bao M Ma R Chao J. Efficiency and equity of pension care service resource allocation in China, 2014-2022. Health Policy Technol. (2025) 14:101016. doi: 10.1016/J.HLPT.2025.101016

[7] Li M Peng P Ao Y Zhou Z Zuo J Martek I. Equity in public decision-making: a dynamic comparative study of urban-rural pension care institution resource allocation in China. Humanit Soc Sci Commun. (2024) 11:1526. doi: 10.1057/s41599-024-04041-x

[8] Zhang L Wei L Zhang W Fang Y. Bridging the gap: coordinating equity and efficiency in older people care resource allocation in China. BMC Geriatr. (2024) 24:165. doi: 10.1186/s12877-024-04696-w, 38365604 PMC10874015

[9] Pavlidis G Hansen T Motel-Klingebiel A Aartsen M. Network and solitude satisfaction as modifiers of disadvantages in the quality of life of older persons who are challenged by exclusion from social relations: a gender-stratified analysis. Appl Res Qual Life. (2022) 17:2859–75. doi: 10.1007/s11482-022-10045-z, 35342486 PMC8934721

[10] Borgatti SP Mehra A Brass DJ Labianca G. Network analysis in the social sciences. Science. (2009) 323:892–5. doi: 10.1126/science.1165821, 19213908

[11] Provan KG Kenis P. Modes of network governance: structure, management, and effectiveness. J Public Adm Res Theory. (2008) 18:229–52. doi: 10.1093/jopart/mum015

[12] Taket AR. Resource allocation problems and health services for older adults. World Health Stat Q. (1992) 45:89–94. 1413862

[13] You N. Spatial disparities in pension care institution accessibility: integrating real-time navigation data and older adults’s preferences in Fuzhou, China. BMC Public Health. (2026) 26:882. doi: 10.1186/S12889-026-26536-9 PMC12983847 41664016

[14] Luan J Tian Y Jim CY Zheng H Fan S. Assessing pension’s perceived accessibility of community hospitals from a satisfaction perspective in China’s Beijing metropolis. Health Policy Technol. (2025) 14:101080. doi: 10.1016/J.HLPT.2025.101080

[15] Gu M Li S Zheng G. Optimizing spatial accessibility and equity of hierarchical older adult care facilities using a multi-modal two-step floating catchment area method: a case study of Lin’an district, Hangzhou. Front Public Health. (2025) 13:1559463. doi: 10.3389/fpubh.2025.1559463, 40337731 PMC12055535

[16] Li M Peng P Zhu H Ao Y Zhou Z Long Y . Assessing the gap: resource efficiency of institutionalised pension care in urban and rural China. Public Health. (2025) 238:45–52. doi: 10.1016/j.puhe.2024.11.017, 39612801

[17] Li M Peng P Zhu H Ao Y Zhou Z. Exploring the equitable spatial planning on the accessibility of institutional pension care in megacity: a study based on Chengdu, China. Sustain Cities Soc. (2024) 117:105998. doi: 10.1016/J.SCS.2024.105998

[18] Sun X Cheng Y Tao Z. Spatial accessibility and equity of residential care facilities in Beijing from 2010 to 2020. Health Place. (2024) 86:103219. doi: 10.1016/j.healthplace.2024.103219, 38467103

[19] Liu B Qiu N Zhang T. Accessibility of pension care facilities based on social stratification: a case study in Tianjin, China. Sustainability. (2023) 15:1507. doi: 10.3390/SU15021507

[20] Chen Y Liu B Shen Y Li L. Assessing accessibility to service facilities for older people in age-restricted communities from the perspective of equity. J Transp Health. (2022) 27:101515. doi: 10.1016/J.JTH.2022.101515

[21] Cheng T Liu C Yang H Wang N Liu Y. From service capacity to spatial equity: exploring a multi-stage decision-making approach for optimizing pension-care facility distribution in the city Centre of Tianjin, China. Sustain Cities Soc. (2022) 85:104076. doi: 10.1016/J.SCS.2022.104076

[22] Ravensbergen L Van Liefferinge M Jimenez I Zhang M El-Geneidy A. Accessibility by public transport for older adults: a systematic review. J Transp Geogr. (2022) 103:103408. doi: 10.1016/J.JTRANGEO.2022.103408

[23] Guida C Carpentieri G Masoumi H. Measuring spatial accessibility to urban services for older adults: an application to healthcare facilities in Milan. Eur Transp Res Rev. (2022) 14:23. doi: 10.1186/s12544-022-00544-3, 38625259 PMC9160510

[24] Deville-Stoetzel N Kaczorowski J Agarwal G Lussier M-T Girard M. Participation in the cardiovascular health awareness program by older adults residing in social housing in Quebec: social network analysis. BMC Health Serv Res. (2021) 21:37. doi: 10.1186/S12913-020-06019-2 PMC7791708 33413324

[25] Yang Y Liang X Liu Q Liu J. Navigating limited resources: experiences of caregivers for pension terminal cancer patients in a region with limited palliative care services. Support Care Cancer. (2025) 33:207. doi: 10.1007/s00520-025-09270-7 39971822

[26] Zheng F Tang X Zhang X. Analysis of daily life, spiritual comfort, and fall safety care needs of older population in urban and rural areas. J Clin Nurs Res. (2025) 9:157–63. doi: 10.26689/jcnr.v9i1.9497

[27] Yang E. Study on the impact of children’s economic support on social participation of urban and rural older population: empirical research based on CHARLS. Acad J Manag Soc Sci. (2024) 7:38–43. doi: 10.1007/S00520-025-09270-7

[28] Aung TNN Aung MN Moolphate S Koyanagi Y Ichikawa M Supakankunti S . Estimating service demand for intermediary care at a community integrated intermediary care center among family caregivers of older adults residing in Chiang Mai, northern Thailand. Int J Environ Res Public Health. (2021) 18:6087. doi: 10.3390/ijerph18116087, 34200048 PMC8200191

[29] Kim H Kwon S Kim S-M. The effects of patient and facility characteristics on the resource use by older adults in long-term care services. Health Policy Manag. (2002) 12:21–53. doi: 10.54097/E5XPQ251

[30] Roy JR Thill J-C. Spatial interaction modelling. Pap Reg Sci. (2004) 83:339–61. doi: 10.1007/s10110-003-0189-4

[31] Chen Y. The distance-decay function of geographical gravity model: power law or exponential law? Chaos Solitons Fractals. (2015) 77:174–89. doi: 10.1016/j.chaos.2015.05.022

[32] Fotheringham AS. Spatial structure and distance-decay parameters. Ann Assoc Am Geogr. (1981) 71:425–36. doi: 10.1111/j.1467-8306.1981.tb01367.x

[33] Merlin LA. A new method using medians to calibrate single-parameter spatial interaction models. J Transp Land Use. (2020) 13:49–70. doi: 10.5198/jtlu.2020.1614

[34] Wu Y Tan W Yi W Chen Y. Enhancing pension care services in high-density aging communities: a dual-dimensional GIS-SPO framework for spatial and temporal optimization. Int J Health Geogr. (2025) 24:44. doi: 10.1186/s12942-025-00437-3 PMC12723929 41345664

[35] Li H Sun S Bartomeu-Magaña E Wei X. Determinants of community-based home care service demand among urban older adults in Shanxi, China: a cross-sectional psychological perspective. Front Public Health. (2025) 13:1645632. doi: 10.3389/fpubh.2025.1645632 PMC12417115 40933403

[36] Alsaeed T Washington T Xia B. The kingdom’s emerging pension care landscape: insights for the future of elder care in Saudi Arabia—a systematic review. Front Public Health. (2026) 14:1825664 10.3389/fpubh.2026.1825664 PMC13343352 42422669

